# Chip-based ion chromatography (chip-IC) with a sensitive five-electrode conductivity detector for the simultaneous detection of multiple ions in drinking water

**DOI:** 10.1038/s41378-020-0175-x

**Published:** 2020-08-24

**Authors:** Xiaoping Li, Honglong Chang

**Affiliations:** Ministry of Education Key Laboratory of Micro/Nano Systems for Aerospace, School of Mechanical Engineering, Northwestern Polytechnical University, 710072 Xi’an, P. R. China

**Keywords:** Engineering, Chemistry

## Abstract

The emerging need for accurate, efficient, inexpensive, and multiparameter monitoring of water quality has led to interest in the miniaturization of benchtop chromatography systems. This paper reports a chip-based ion chromatography (chip-IC) system in which the microvalves, sample channel, packed column, and conductivity detector are all integrated on a polymethylmethacrylate (PMMA) chip. A laser-based bonding technique was developed to guarantee simultaneous robust sealing between the homogeneous and heterogeneous interfaces. A five-electrode-based conductivity detector was presented to improve the sensitivity for nonsuppressed anion detection. Common anions (F^−^, Cl^−^, NO_3_^−^, and SO_4_^2−^) were separated in less than 8 min, and a detection limit (LOD) of 0.6 mg L^−1^ was achieved for SO_4_^2−^. Tap water was also analyzed using the proposed chip-IC system, and the relative deviations of the quantified concentration were less than 10% when compared with that a commercial IC system.

## Sensors: Detecting ion concentrations in drinking water

Researchers in China have developed a new system to measure the concentration of ions in water to ensure that it is safe to drink. While ions can be easily detected with simple color-strip tests, quantitative measurement currently requires ion chromatography in a lab. Honglong Chang’s team at Northwestern Polytechnical University designed a device which uses chip-based ion chromatography using five electrodes to improve the signal-to-noise ratio. The team tested their device by using it to measure the concentration of several anions in tap water and found that it performed comparably to existing commercial ion chromatography systems.

## Introduction

Safe drinking water is a critical resource for human health^1,2^. Among a variety of contaminants, some chemical species present an ongoing concern^3^. For example, an epidemiological study has shown that long-term exposure to fluoride and nitrate in drinking water can lead to chronic diseases^4^. In addition, chloride and sulfate may cause certain aesthetic effects. Therefore, the allowed concentrations of these species in drinking water have been regulated worldwide (Table 1). It is of great health significance for end-users to measure the concerned ions accurately, inexpensively, and conveniently.

**Table 1:** Maximum contaminant levels (MCLs, in mg L^−1^) for concerned anions according to drinking water quality standards by the World Health Organization (WHO), European Union (EU), United States of America (USA), and China

	WHO	EU	USA	China
Fluoride (F^−^)	1.5	1.5	4.0	1.0
Nitrate (NO_3_^−^)	50	50	10 (as N)^a^	10 (as N)^a^
Chloride (Cl^−^)	–	250	250	250
Sulfate (SO_4_^2−^)	–	250	250	250

Dash (–) indicates that Guideline values have not been established

^a^10 mg L^−1^ (NO_3_^−^ as N) ≈ 44.3 mg L^−1^ (NO_3_^−^)

Although the foregoing anions can be measured on-site by using simple commercial test kits based on color reactions on specific strips (Table S1 and Fig. S1), only qualitative results can be obtained, and only a single element can be detected in one test. In contrast, anions can be quantitatively and simultaneously determined in a laboratory by ion chromatography (IC)^5^, which is considered the standard method for detecting common cations and anions in water samples. Unfortunately, current chromatography systems are too bulky for use in field tests^6,7^. In past decades, advancements in lab-on-a-chip technology have successfully addressed this issue for both chip-based gas chromatography (chip-GC)^8–11^ and chip-based liquid chromatography (chip-LC)^12–15^ but relatively less so for chip-IC. Kidd et al.^16^ packed a silicon microchannel with anion resin to test common anions by incorporating an external syringe pump, injector, and contactless conductivity detector (C^4^D). Tanaka et al.^17^ presented a polymethylmethacrylate (PMMA) chip packed with anion exchangers to detect glycated hemoglobin (HbA_1c_). However, the chip was also operated by a commercial syringe pump, valve, and electrochemical detector. Previously, our group also reported a packed PMMA IC chip for the detection of HbA_1c_^18^. Ultraviolet–visible spectroscopy (UV/Vis) detection was performed through a fiber-coupled microspectrometer. Despite these advances, developing a compact anion-oriented chip-IC is still a challenge. On the one hand, the current chip-IC mainly relies on discrete commercial parts, and the level of integration needs to be further improved. On the other hand, a lightweight but sensitive on-chip detector for anions should also be developed.

Basically, the primary parts of a chip-IC system comprise a micropump, injector, column, and detector. Considering that band broadening only occurs at the flow path between the sample injection channel, column, and detection cell^12^, the integration of a micropump is nonessential, and a traditional LC pump or syringe pump can still be used for chip-IC. Nevertheless, an integrated micropump can dramatically reduce the size and weight of the whole system^19^.

The column is the most important part of an IC system. Though on-chip columns have been achieved in open-tubular^20,21^, monolithic^22,23^, and pillar array^24,25^ forms, it is straightforward to pack the channel with commercially available resins, and better reproducibility can be guaranteed because no surface modification of the particulates is needed^26^. To keep beads in the channels, frit structures with feature sizes smaller than the resin’s diameter are usually needed. However, commonly used frits, such as weirs^27^, step-shaped channels^28^, and parallel small channels^29^, follow a similar multistep lithography and etching process, and interfacial bonding is also severely challenged by the high-pressure resistance of the packed bed. Therefore, more attention should be given to both the chip material and corresponding fabrication. For example, the commonly used polydimethylsiloxane (PDMS) in microfluidics is limited in chip-LC because PDMS is gas permeable and the bonding strength is relatively poor^30^.

Sample injection on-chip has been mainly achieved by using T-shaped^31,32^ or cross-shaped^27,33^ channels. However, external valves are needed for fluid on/off control at the sample inlet and outlet. In contrast, integrated microvalves are urgently desired^34^, but their implementation is restricted by the high working pressure^35^ or until the poor bonding strength of the heterogeneous interface between the substrate and valve membrane is improved.

Ultrasensitive detectors, such as mass spectrometry (MS)^22,28,29,36^ and laser-induced fluorescence (LIF)^20,21,23^, have been widely used in chip-LC to measure biomolecules, organic compounds, and pharmaceuticals. However, conductivity detection (CD) is the first choice for IC due to its universality to ionic samples^37^. In macro-IC systems, a suppressor is commonly used to neutralize the high background conductivity of the eluent before CD to increase the detection sensitivity. Although a suppressor on-chip system has recently been reported^38^, the integration of suppressors in a chip-IC system is much more complicated; therefore, nonsuppressed CD, which uses a low-conductivity eluent for direct CD after separation, is more suitable for chip-IC. In terms of the detector cell, electrodes can be either in direct contact or not with the eluate. Although the contact mode can provide higher sensitivity than the contactless model^39^, the electrode reaction may lead to large baseline noise and thus compromise the signal-to-noise ratio (S/N) and sensitivity. Therefore, a contact CD with steady and reduced baseline noise is helpful to increase the sensitivity in nonsuppressed chip-IC.

Herein, we developed a compact chip-IC system for the detection of anions in drinking water. A channel network with various cavities was micromilled in one step on a PMMA substrate. Commercial sintered frits and valve membrane disks were embedded into the corresponding cavities for integration. Both homogeneous and heterogeneous interfaces were robustly sealed by laser bonding to resist the high working pressure. A conductivity detector with a five-electrode configuration was integrated to improve the S/N in the nonsuppressed mode. Separation of both anion standards and tap water was demonstrated by using the proposed chip-IC system.

## Results and discussion

### Chip-IC system

**Fig. 1:**
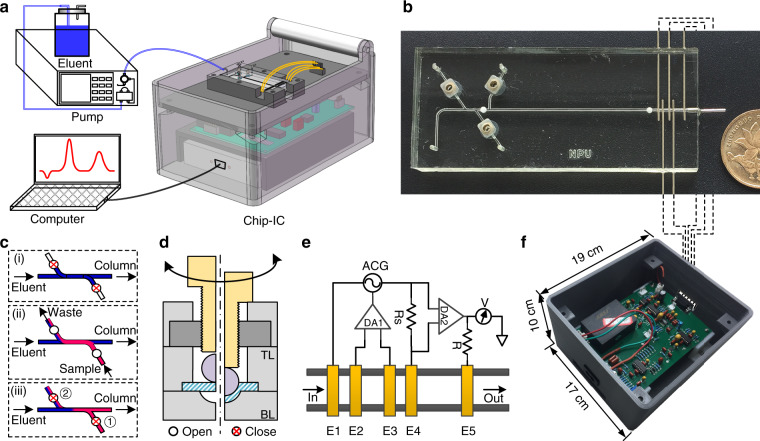
Schematic of the chip-IC platform. **a** Chip-IC-based experimental setup. **b** Photo of a fabricated IC chip. **c** On-chip valve-based sample injection procedure: (i) baseline stabilization, (ii) sample loading, and (iii) sample injection. **d** Principle of valve control. **e** Principle of five-electrode conductivity detection. **f** Image of the circuit in the chip-IC box. The five electrodes are connected to the serial port on the circuit for signal transmission

Figure 1a presents an overview of the experimental setup. The chip-IC system consists of an IC chip as well as necessary electronics including a data processing circuit and a data acquisition (DAQ) unit, and it is arranged in a 3D-printed box that is 17 × 19 × 10 cm^3^ in size and less than 2 kg in weight. A conventional pump is connected to the chip through PEEK fitting (Agilent, Beijing, China) for eluent delivery. The chromatographic signal is read from the DAQ and recorded on a computer. For chromatographic separation, the IC chip (Fig. 1b) uses the sample injection procedure shown in Fig. 1c. Briefly, the packed channel is first equilibrated using the eluent to obtain a steady baseline. The two valves are then opened, and the sample is injected from the inlet. After injection, the two valves are closed sequentially, and the sample plug will be drawn into the packed channel for separation. All valves are currently driven by screw^40,41^ (Fig. 1d); however, micromotors or solenoid valves can also be incorporated for automated control^40^. To obtain an exact on/off operation under high pressure, a tiny ball is adopted to force the membrane to be in close contact with the hemispheric valve chamber.

Figure 1e shows the principle of the five-electrode CD system. The five electrodes include the two reference electrodes (E1 and E4), two detection electrodes (E2 and E3), and an electrical ground electrode (E5). A sinusoidal current is applied to E1 and E4 (denoted as I_14_) and is controlled by a variable AC generator (ACG). E2 and E3 are inputs to a differential amplifier (DA1). When the conductance between E2 and E3 (G_23_) changes, the output of DA1 will force the ACG to vary I_14_ to maintain a constant potential drop between E2 and E3 (U_23_). In response, the potential drop on the sampling resistor (U_Rs_) will also vary and be further converted by another differential amplifier (DA2) and finally detected by voltmeter V. E5 reduces the capacitance leakage to the ground. Because there is only a very low current at E2 and E3, common problems in contact mode, such as Faradaic impedance, are largely eliminated. Therefore, the 5e detector will give a more stable baseline and accurate measurement of the solution conductance, which makes it very suitable for nonsuppressed IC detection^42^. A developed PCB for signal processing is presented in Fig. 1f.

### Chip fabrication

**Fig. 2:**
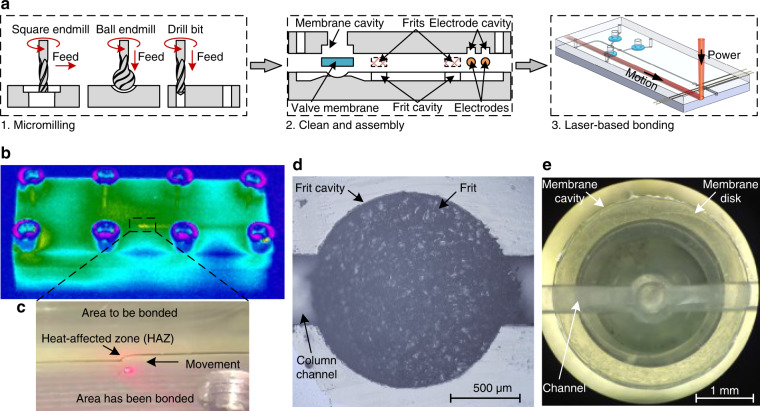
Chip fabrication. **a** Schematic of the fabrication process: (1) micromilling to prototype single-layered chips; (2) chips cleaned and assembled; and (3) laser bonding to seal the interface. **b** Infrared photograph of the laser-bonded chip with **c** an enlarged view of the heat-affected zone (HAZ). Photograph of the embedded **d** frit and **e** valve membrane

The device is fabricated by combining micromilling and laser bonding techniques. The process (Fig. 2a) mainly involves three steps: (1) single-layered chips with various channel geometries are micromilled^43^; (2) small chips are cleaned, and a pair of pieces as well as frits, valve membranes, and electrodes are assembled together; and (3) the assembled block is laser-bonded (Fig. S2).

From the infrared view (Fluke Ti400, Beijing, China) of laser bonding (Fig. 2b), only a small local zone of PMMA is heated to high temperature and melts (Fig. 2c). The PMMA melts can slightly flow into any interfacial gaps, and after resolidification, effective sealing is achieved on both the homogeneous PMMA interface and heterogeneous interfaces, e.g., PMMA/frit, PMMA/valve membrane, and PMMA/electrode (Fig. 2d, e). The normal stress of bonded chips was measured using a tensile tester (SFMIT, Suzhou, China). Dumbbell-shaped specimens with a 1.5 × 2 mm^2^ cross-section were tested at a pull rate of 1.0 mm min^−1^ (Fig. S3). Most (6 of 8) of the specimens were fractured out of the bonded interface, and the average normal strength was calculated to be 74.5 MPa, with a relative standard deviation (RSD) of 0.64% (*n* = 8). This value is much larger than that reported by Hsu et al.^44^ (17.6 MPa) and Tran et al.^45^ (6.17 MPa). Sealing between heterogeneous interfaces was tested by using the burst pressure method (Fig. S4). A burst pressure of 10.6 MPa was recorded with leakage occurring at the world-to-chip connection rather than the chip interface. This value is also much higher than that reported by Hsu et al.^44^ (0.69 MPa) and is sufficient for column packing and chromatographic separation.

### Column packing quality

**Fig. 3:**
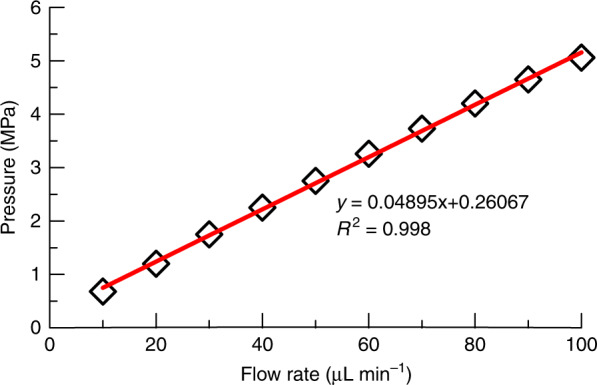
Pressure flow-rate curve for the on-chip packed channel. Data was measured (open diamond) with DI water as the working fluid and linear fitted (line).

The chip was packed using a slurry method (Fig. S5). The dimensionless flow resistance *ϕ* gives a comparative measure of the packing quality of a column, which is defined as^46^

1 $$\frac{\phi }{\varepsilon } = \frac{{d_{\mathrm{p}}^2A_{\mathrm{c}}}}{{\eta L_{\mathrm{c}}}}\frac{{\Delta p}}{F}$$

where *ε* is the porosity of the column (typically ~0.65), ∆*p* is the pressure drop of the column, *d*_p_ is the bead diameter, *A*_c_ is the column cross-sectional area, *L*_c_ is the column length, and *η* and *F* are the viscosity and flow rate of the mobile phase, respectively. Generally, *ϕ* is 500–1000 for packed columns, and a lower value usually indicates poor packing with voids or cracks. Figure 3 shows the pressure drop at varying flow rates of DI water, where *ϕ* is calculated to be 650. This value is well within the typical range of 500–1000, meaning that a favorable packed quality was achieved. In addition, according to the *ϕ* value of 355 with assumed *ε* = 0.8 obtained in a parylene channel packed with the same beads used here^46^, *ϕ* is calculated to be 289 with *ε* = 0.65. The higher *ϕ* values here also mean that better packing quality was achieved by following the current column design and packing technique.

### Detection of anion standards

**Fig. 4:**
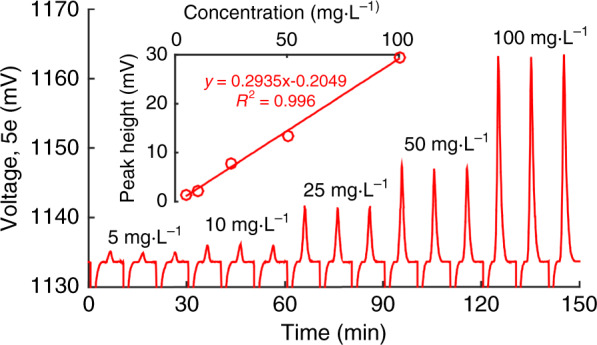
Chromatograms of SO_4_^2−^ standards tested at a flow rate of 25 μL min^−1^ (*n* = 3). The inset shows the calibration plot. Eluent: 4-mM *p*-HBA containing 2.5% methanol (pH=8.5). Injection: 0.5μL. Detection: ambient

A set of SO_4_^2−^ standard solutions with concentrations of {5, 10, 25, 50, and 100 mg L^−1^} were tested at a flow rate of 25 μL min^−1^, with each concentration tested three times. As shown in Fig. 4, good linearity (*R*^2^ = 0.996) was observed in the range of 5–100 mg L^−1^ for the 5e detector. The RSDs of the retention time *t*_R_ and peak height *H* are 0.2–0.5% and 0.6–2.4%, respectively. Based on the observed S/N at 5 mg L^−1^, the S/N = 3 LOD is calculated to be 0.6 mg L^−1^.

**Fig. 5:**
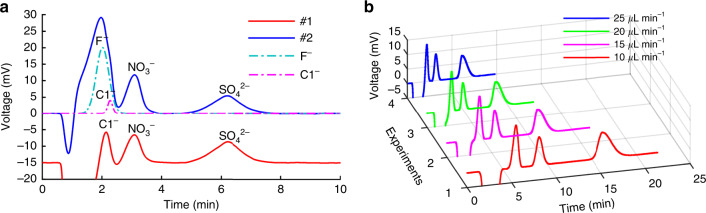
Chromatograms of standard mixtures. **a** Mixture #1 (10–20–20) and mixture #2 (50–10–30–20) both tested at a flow rate of 25 μL min^−1^. **b** Mixture #1 (10–20–20) tested at varying flow rates. Dashed lines in **a** represent peaks of F^−^ and Cl^−^ discriminated from the overlapping curve of mixture #2. Other conditions are the same as in Fig. 4

Two standard mixtures, i.e., (#1, 10–20–20) containing 10 mg L^−1^ Cl^−^, 20 mg L^−1^ NO_3_^−^, and 20 mg L^−1^ SO_4_^2−^ and (#2, 50–10–30–20) containing 50 mg L^−1^ F^−^, 10 mg L^−1^ Cl^−^, 30 mg L^−1^ NO_3_^−^, and 20 mg L^−1^ SO_4_^2−^, were tested using the packed chip at a flow rate of 25 μL min^−1^. As shown in Fig. 5a, F^−^ and Cl^−^ overlap under the current chip and conditions. This overlap may be due to the large water dip in nonsuppressed IC, which has been confirmed in nonsuppressed capillary ICs^47^. However, the overlapping peaks of F^−^ and Cl^−^ can be discriminated by postprocessing in software such as OriginPro (Fig. S6). On the other hand, it is very promising to separate F^−^ and Cl^−^ directly by using more advanced and smaller resins and optimizing the buffer condition.

The three-anion mixture (#1, 10–20–20) was tested at varying flow rates. Figure 5b shows that the smaller flow rate achieves better resolution, although the analysis time is longer. At a flow rate of 25 μL min^−1^, all three ions can be detected within 8 min. The separation efficiency, namely, the number of theoretical plates, is defined as *N* = 5.54(*t*_R_/*W*_h/2_)^2^, where *t*_R_ is the retention time and *W*_h/2_ is the full width at half maximum (FWHM). As the flow rate varies from 25 μL min^−1^ to 10 μL min^−1^, the *N* values (plates m^−1^) for Cl^−^, NO_3_^−^, and SO_4_^2−^ are 3800–7100, 7100–11,200, and 6100–9600, respectively (Table S3). Compared with nonsuppressed CapIC^47^, chip-IC showed better efficiency in the separation of relatively strongly retained NO_3_^−^ (reported *N* = 5400); nevertheless, the *N* value of Cl^−^ in chip-IC is only half that reported (*N* = 15,800) in CapIC. The large difference in relatively weakly retained species may also be attributed to the large water dip in the nonsuppressed IC.

### Detection of tap water

**Fig. 6:**
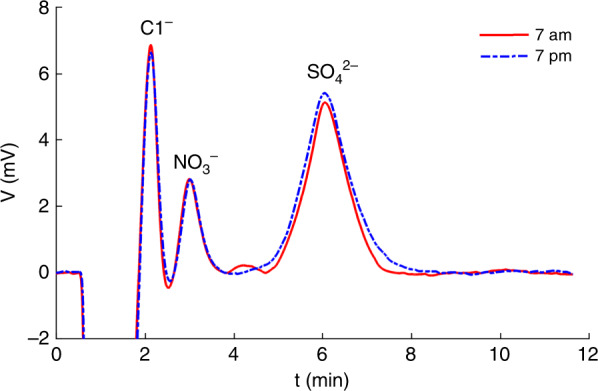
Chromatograms of anions in tap water detected by chip-IC. The conditions are the same as in Fig. 4

Tap water was successfully analyzed using the chip-IC system at room temperature. A water sample was collected directly from the tap, filtered, and then injected for separation. Separation tests were performed at 7 am and 7 pm. Figure 6 and Table 2 show the obtained chromatograms and corresponding concentrations of anions, respectively. Water samples were also tested on a benchtop IC system (IC-8286, Luhai Photoelectric, Qingdao, China), which confirmed that only F^−^, Cl^−^, NO_3_^−^, and SO_4_^2−^ were determined. Therefore, no other overlap occurs on the chromatograph by chip-IC except for F^−^ and Cl^−^. As mentioned, the overlapping F^−^ and Cl^−^ peaks can be post-treated for peak discrimination. However, the concentration of F^−^ is so small that the postprocessing would result in a large relative error. To assess the true detectability of the chip-IC system, only NO_3_^−^ and SO_4_^2−^ data by chip-IC are quantified and presented in Table 2, which reveals that the relative deviations of concentrations of these anions obtained by chip-IC are less than 10% when compared with the commercial IC-8286 system.

**Table 2:** Comparison of detected concentrations of anions in tap water by Chip-IC and IC-8286 (mg L^−1^)

Species	7 am			7 pm		
	Chip-IC	IC-8286	Relative deviation	Chip-IC	IC-8286	Relative deviation
F^−^	–	0.71		–	0.77	
Cl^−^	–	5.4		–	5.8	
NO_3_^−^-N	4.7	4.3	9.3%	4.6	4.4	4.5%
SO_4_^2−^	17.1	16.6	3.0%	20.1	19.6	2.6%

## Materials and methods

### Reagents

Isopropanol, methanol, *p*-hydroxybenzoic acid (*p*-HBA), sodium chloride, sodium hydroxide, anion stock solutions (1000 mg L^−1^) of fluoride (F^−^), chloride (Cl^−^), nitrate (NO_3_^−^), and sulfate (SO_4_^2−^), and two standard mixtures—one containing 10 mg L^−1^ Cl^−^, 20 mg L^−1^ NO_3_^−^, and 20 mg L^−1^ SO_4_^2−^ (#1, 10–20–20), and the other containing 50 mg L^−1^ F^−^, 10 mg L^−1^ Cl^−^, 30 mg L^−1^ NO_3_^−^, and 20 mg L^−1^ SO_4_^2−^ (#2, 50–10–30–20)—were all acquired from Sigma–Aldrich (Shanghai, China) at reagent grade. Single-anion calibration standards were prepared by diluting the stock solution with deionized water to the desired concentration. The eluent was prepared in 4.0 mM *p*-HBA solution containing 2.5% methanol, with the pH adjusted to 8.5 by 0.1 M NaOH. All solutions were filtered using a 0.2 μm filter before use.

### Chip design and fabrication

The device was designed using SolidWorks (Dassault Systemes, Waltham, MA, USA). The whole chip consists of two layers: the bottom layer (BL) containing the microchannel network including a double-T sample injection junction, valve chamber, frit cavities, separation column, and detection cell and the top layer (TL) containing the valve membrane cavities, electrode cavities and through vias for valve control and tube connection. The cross-sectional size of the channel is 500 × 500 μm^2^. A 0.5 µL sample plug is defined by a double-T-shaped side channel at a distance of 2 mm. The separation channel is 40 mm long. Two frit cavities are set at each side of the separation channel, and the depth and diameter of each frit cavity are 0.5 mm and 1.4 mm, respectively. The diameter of commercially available ultrahigh-molecular weight polyethylene (UHMW-PE) frits (Biocomma, Shenzhen, China) is also 1.4 mm. Another side channel is set to introduce the slurry for chip packing. Every side channel has a membrane microvalve integrated for fluid on/off manipulation. The diameter and depth of the spherical valve chamber are 0.5 mm and 0.6 mm, respectively. The depth and diameter of valve membrane cavities are 4 mm and 0.5 mm, respectively; 4-mm-diameter disks are cut from a 500-µm-thick highly elastic silicone sheet (Sanhe 3A Rubber & Plastic Co., Hebei, China) to function as valve membranes. The diameters of the through vias for valve control and tube connection are 1 mm and 3 mm, respectively. The total chip size is 30 × 70 mm^2^.

Additionally, 3-mm-thick PMMA sheets (Evonik Röhm, Darmstadt, Germany) are micromilled to create channels and then cut into pieces using a CNC machine (Jingdiao, Beijing, China). Small pieces are ultrasonically cleaned in isopropanol for 2 min, rinsed in DI water, dried with nitrogen, and then laser-bonded at room temperature. Briefly, the Clearweld™ absorber (Crysta-Lyn, Binghamton, NY, USA) is first manually coated on the top surface of the BL. Then, the frits and membrane disks are inserted into the frit cavities and membrane cavities. Pt wires 0.5 mm in diameter are inserted into the electrode channels. Then, a pair of chips are stacked, clamped using a homemade fixture, and laser-bonded. The focused spot is ~400 μm, and the optimal laser power, scanning velocity and pitch are 10 W, 8 mm s^−1^, and 0.4 mm, respectively.

### Column packing

An approximately 80 mg mL^−1^ slurry of 7 µm PRP-X110 anion exchanger (Hamilton Reno, NV, USA) is prepared in a 2 M NaCl solution and ultrasonicated for 1 min. A PEEK tube (0.5 mm i.d. and 10 cm long) filled with slurry is connected between the packing inlet and an HPLC pump (Hanbon Sci. & Tech., Huai’an, China). The channel is packed at a flow rate of 200 μL min^−1^, which is ten times larger than that for separation. The frit has an average pore size of 2 μm and, thus, can keep the beads within the channel. The maximum pumping pressure is set to 10 MPa, at which point the pump will turn off automatically, the packing valve will close, and the packed channel will depressurize spontaneously.

## Conclusions

In this paper, a high-pressure-compatible ion chromatography chip was fabricated by using micromilling and laser-based bonding methods. Owing to the melting–resolidification transition, high bonding strength has been achieved. Using the integrated five-electrode conductivity detector, standard anion solutions and tap water were successfully analyzed. Although cations have not yet been tested, theoretically, better results can be obtained using the same system. With further improvements in micropump integration and automated valve control, we believe that a fully portable chip-IC device would be an alternative tool for field environmental testing, especially for high-throughput detection in water.

## Supplementary information

Supplemental material
