# Supplementary material for: Chip-based ion chromatography (chip-IC) with a sensitive five-electrode conductivity detector for the simultaneous detection of multiple ions in drinking water
Source: Microsyst Nanoeng. 2020 Aug 24;6:66. doi: 10.1038/s41378-020-0175-x (PMC8433475; doi:10.1038/s41378-020-0175-x)
Supplement: Supplementary file 1 — Supplemental material [file 41378_2020_175_MOESM1_ESM.pdf]

# Supplementary Information

## Chip-based ion chromatography (chip-IC) with sensitive five-electrode conductivity detector for simultaneous detection of multi-ions in drinking water

Xiaoping Li[1] and Honglong Chang[1]*

[1]Ministry of Education Key Laboratory of Micro/Nano Systems for Aerospace, School of Mechanical Engineering, Northwestern Polytechnical University, Xi'an 710072, P. R. China

*E-mail: changhl@nwpu.edu.cn

Contents

**Section 1: Commercial water test kits** ........ 1

**Section 2: Laser-based bonding system** ........ 3

**Section 3: Assessment of bonding strength** ........ 4

**Section 4. Chip packing** ........ 6

**Section 5. Performance evaluation** ........ 7

## Section 1: Commercial water test kits

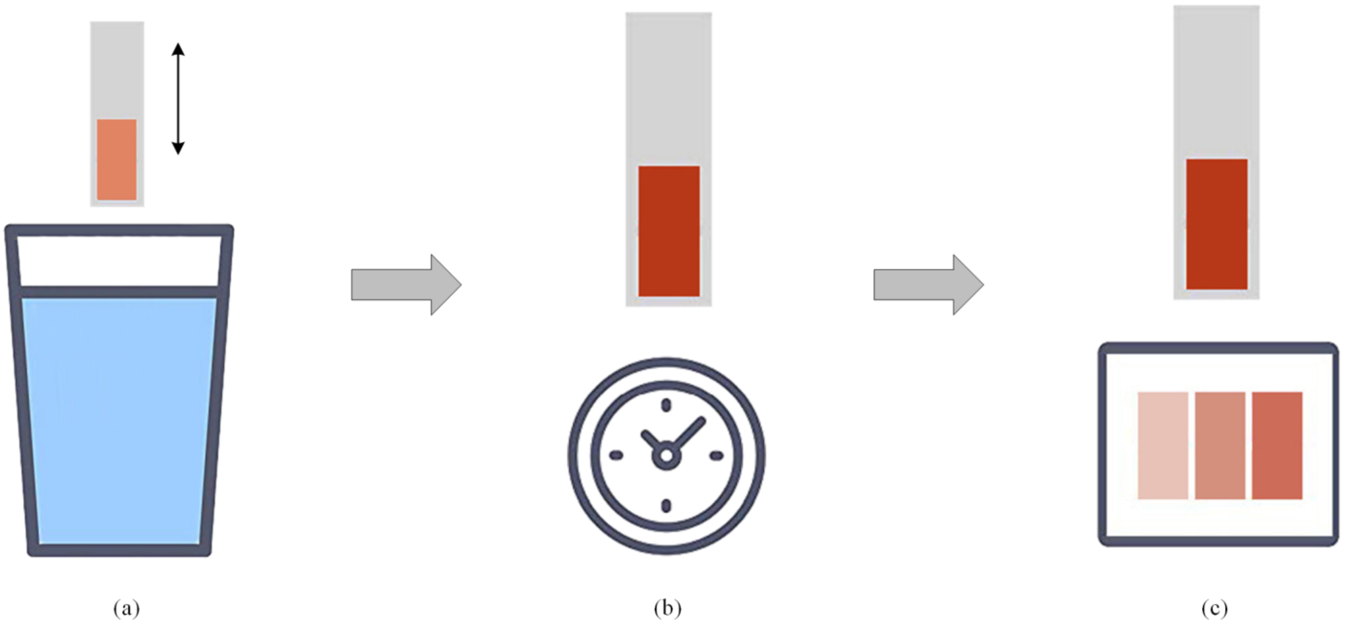


**Figure S1.** Procedure of field kits-based water quality test: (a) dip strip into water sample for 5 seconds, (b) hold strip flat for 30 seconds, and (c) compare strip colour with the colour chart. Only qualitative result can be obtained by using field test kits.

**Table S1.** Representative field drinking water test kits (data from Amazon, access on Dec 15, 2019)

| No | Products | Test items | Cost | Rating (/5) |
|---|---|---|---|---|
| 1 | *Baldwin Meadows* Drinking Water Test Kit | Lead, Iron, Copper, Fluoride, Chlorine, Bromine, Carbonate, Nitrate/Nitrite, Cyanuric Acid, pH, Alkalinity, Hardness | $17.99 | 3.6 |
| 2 | *Watersafe* WS425B Drinking Water Test Kit | Bacteria, Pesticides, Lead, Chlorine, Nitrate/Nitrite, pH, Hardness | $19.95 | 3.7 |
| 3 | *JNW Direct* Drinking Water Test | Lead, Iron, Copper, Chlorine, Nitrate/Nitrite, pH, Alkalinity, Hardness | $19.98 | 3.8 |
| 4 | *First Alert* WT1 Drinking Water Test Kit | Bacteria, Pesticides, Lead, Chlorine, Nitrate/Nitrite, pH, Hardness | $19.99 | 3.7 |
| 5 | *Water-W* Drinking Water Test Strips | Chromium/Cr(VI), Lead, Mercury, Copper, Iron, Fluoride, Chlorine, Bromine, Nitrate/Nitrite, Sulphite, pH, Alkalinity, Hardness | $19.99 | 4.0 |
| 6 | *Crystal Water Filters* Drinking Water Test Kit | Lead, Iron, Copper, Fluoride, Chlorine, Chloride, Bromine, Nitrite/Nitrate, Carbonate, Cyanuric Acid, Ammonium, pH, Alkalinity, Hardness | $19.99 | 4.4 |
| 7 | *Health Metric* Water Test Kit | Bacteria/Coliform, Nitrate/Nitrite, Manganese, Hydrogen Sulphide, pH, Alkalinity, Hardness | $24.97 | 4.2 |
| 8 | *NovoBlue* Home Water Test Kit | Bacteria, Lead, Iron, Copper, Chlorine, Chloride, Nitrate/Nitrite, Sulfate, Hydrogen Sulphide, pH/TA/TCl/TH, | $24.98 | 4.2 |
| 9 | *PurTest* Home Drinking Water Test Kit | Bacteria, Pesticides, Lead, Copper, Iron, Chlorine, Nitrate/Nitrite, pH, Alkalinity, Hardness | $29.95 | 3.7 |
| 10 | *Test Assured* Drinking Water Test Kit | Bacteria, Pesticides, Lead, Iron, Copper, Chlorine, Nitrate/Nitrite, pH, Alkalinity, Hardness | $34.95 | 4.0 |

## Section 2: Laser-based bonding system

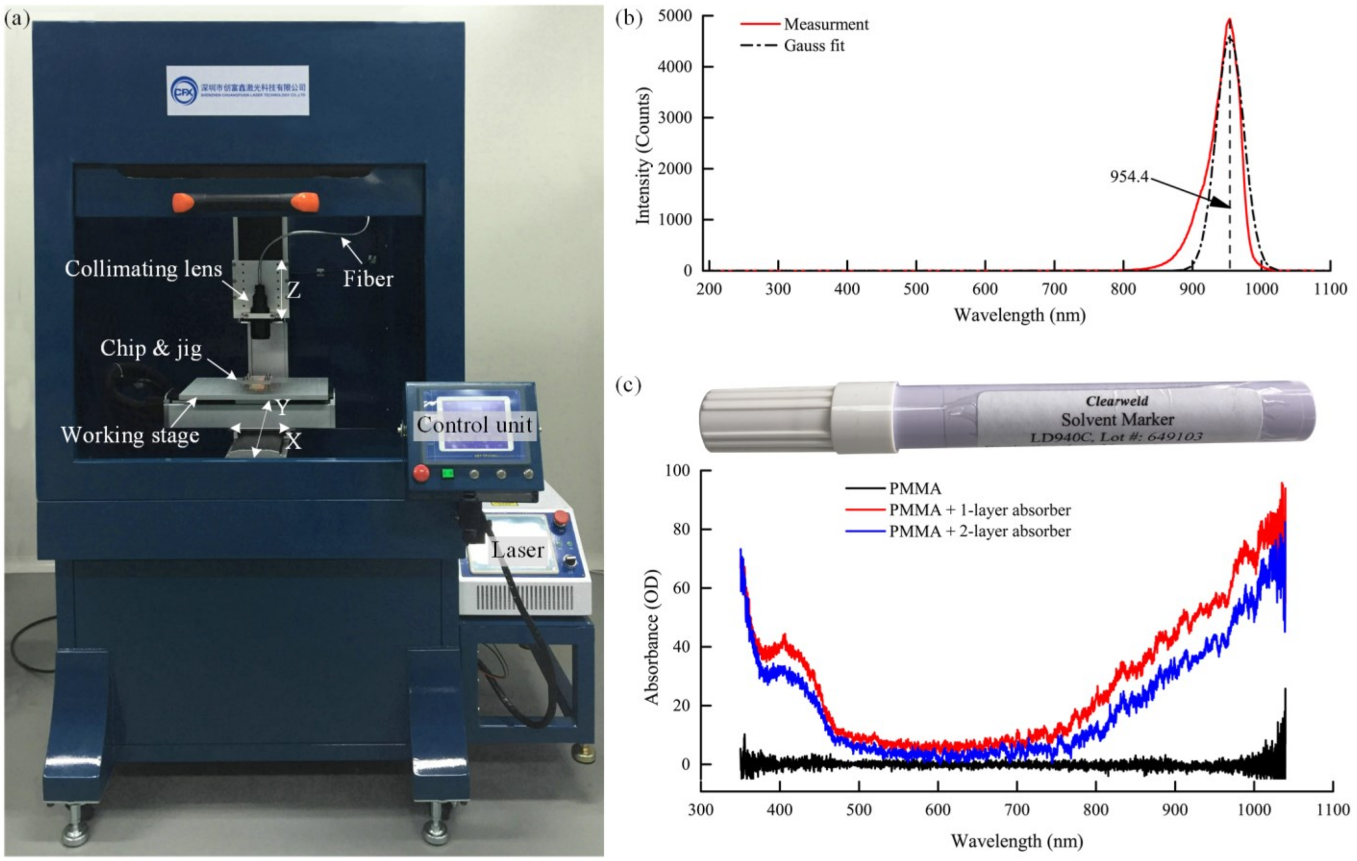


**Figure S2.** Laser-based PMMA bonding: (a) the bonding system, (b) output wavelength of the *LWD40B* laser unit, and (c) the commercially available *Clearweld* absorber and its corresponding absorption spectrum.

The laser-based bonding system (Chuangfuxin, Shenzhen, China) consists of a LWD40B semiconductor laser unit and a precisely mechanical displacement platform. The assembled block is fixed on the working stage with homemade jig, and the output laser beam is transferred by optical fiber and then focused on to the bonded interface by a collimating lens. The movements of the working stage in XY plane as well as the lens in Z direction are precisely programmed by a control unit, with a minimum step displacement of 0.5 µm. The total working range is 200 mm × 130 mm × 75 mm.

The central wavelength of the laser unit is 954.4 nm, and the power can vary from 040 W. According to the supplier, the transmittance of pure PMMA to 950 nm laser is 92%; therefore, a commercially available absorber is adopted to treat the pure PMMA/PMMA interface to dramatically increase the absorbance of 950 nm laser beam.

## Section 3: Assessment of bonding strength

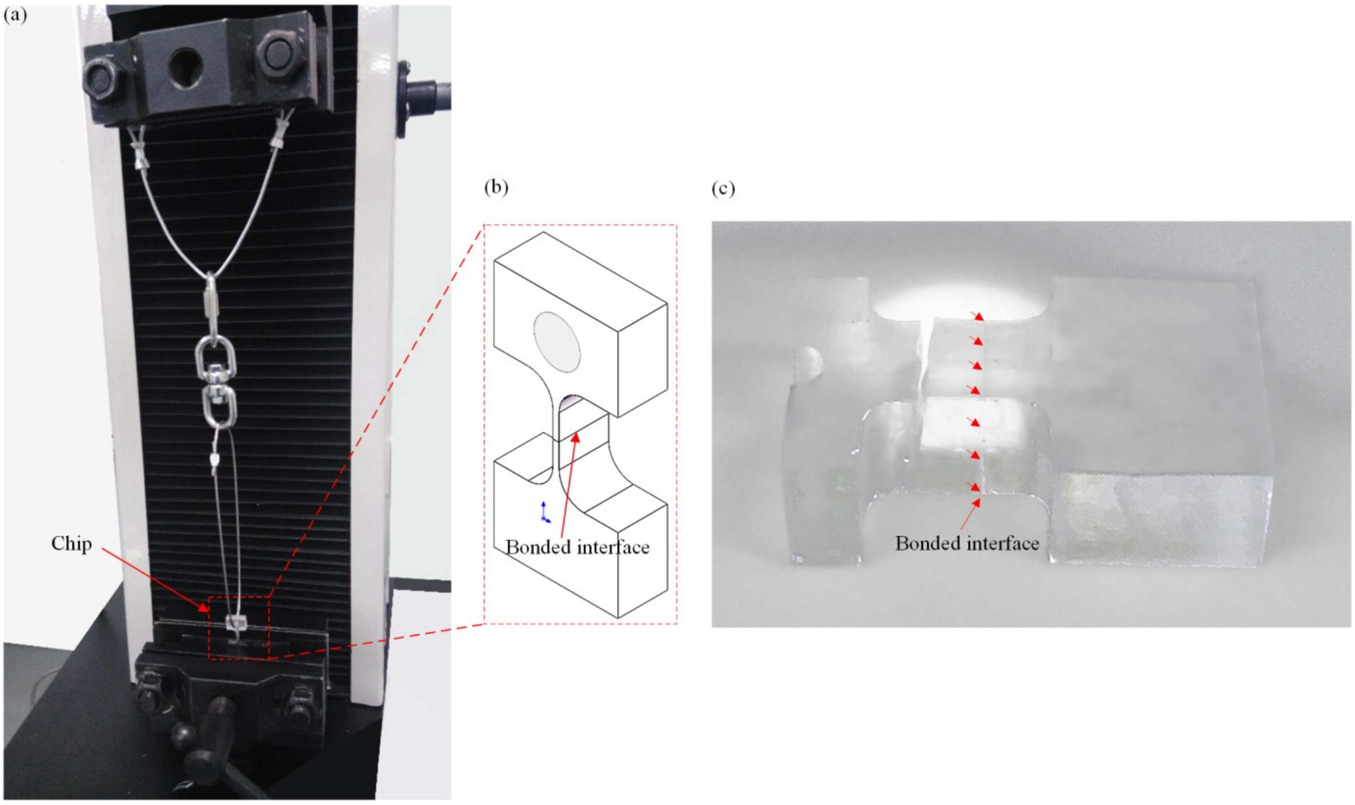


**Figure S3.** (a) Tensile tester with chip mounted. (b) Schematic draw of the tested specimen. (c) Photo of a fractured specimen.

The normal stress of bonded chip was measured using a tensile tester (SFMIT, Suzhou, China). Specimens are cut into dumbbell-shaped with a cross-section of 1.5 mm × 2 $mm^2$. One side of the specimen is directly clamped to the bottom fixed jig. In order to eliminate the transverse shear stress, a whole was milled on the other side, and then connected to the mobile jig by using a flexible steel rope. Specimen is tested at a pull rate of 1.0 $mm \cdot min^{-1}$. Most (6 out of 8) of the specimen were fractured out of the bonded interface. Table S2 record the maximum forces and the corresponding tensile strength calculated by:

$$\sigma = \frac{F}{A}$$

where $\sigma$ is the normal stress, $F$ is the tensile force and $A$ is the area of the cross section.

**Table S2.** Tensile force and corresponding strength.

| No. | 1 | 2 | 3 | 4 | 5 | 6 | 7 | 8 |
|---|---|---|---|---|---|---|---|---|
| $F$ (N) | 223.4 | 222.5 | 225.3 | 220.6 | 223.6 | 223.7 | 224.8 | 223.3 |
| $\sigma$ (MPa) | 74.5 | 74.2 | 75.1 | 73.5 | 74.5 | 74.6 | 74.9 | 74.4 |
| Average | 74.5 MPa (RSD = 0.64%) | | | | | | | |

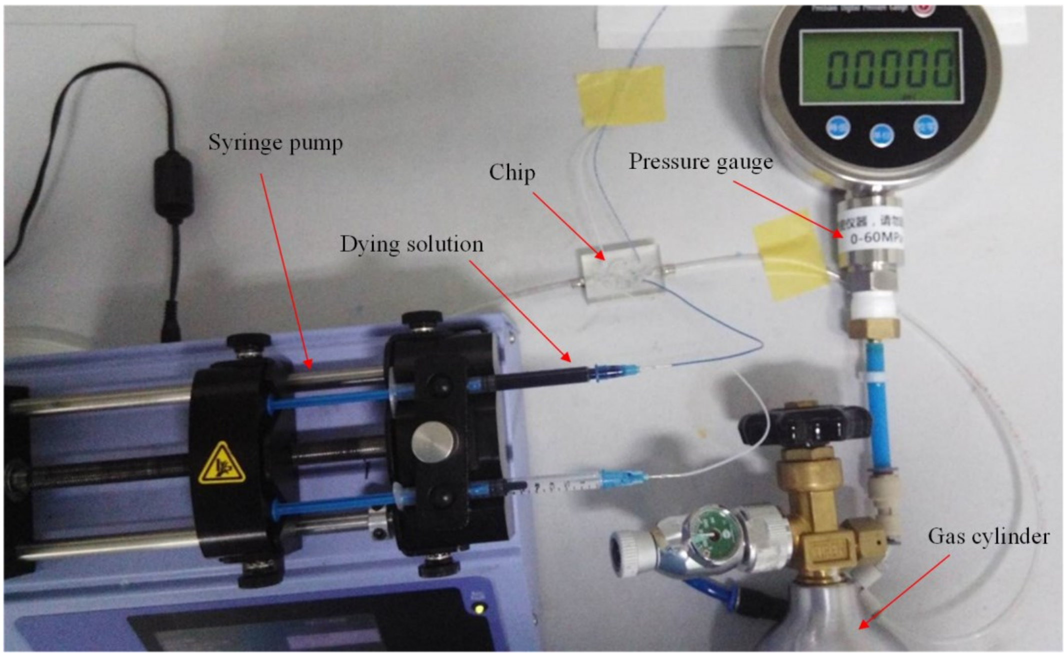


**Figure S4.** Apparatus for burst pressure testing.

The chip is filled with dye solution in advance, and then connected to a $N_2$ gas cylinder, a regulator and a pressure gauge. The cylinder can provide a maximum pressure of 3,000 psi. With valves closed, pressure is gradually increased until leakage occurred. This pressure is recorded as the burst pressure.

**Section 4. Chip packing**

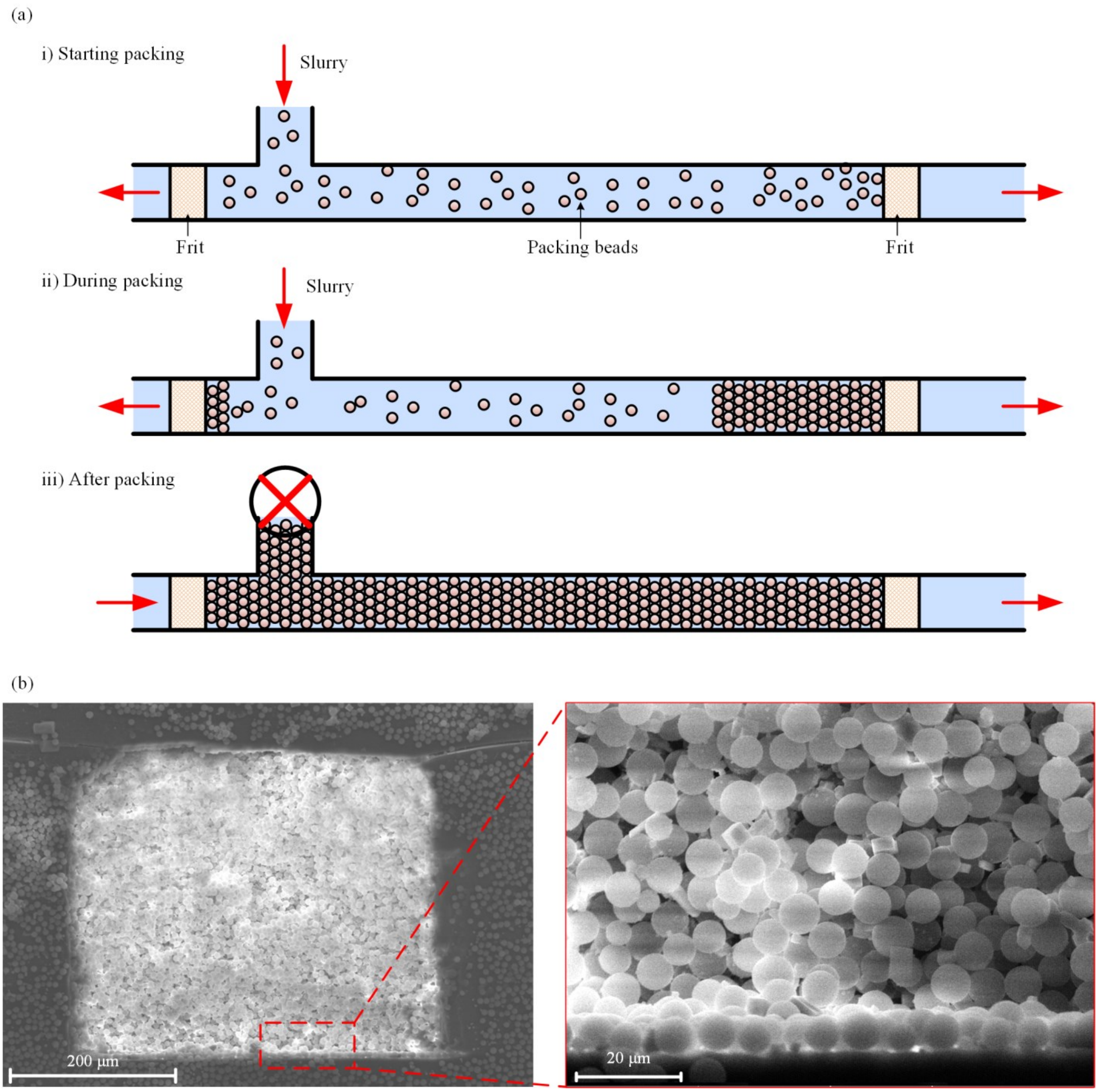


**Figure S5.** Beads-based chip packing. (a) Schematics of the morphologies of the beads inside the channel, and (b) SEM images of the cross-sectional resin-packed beds.

With the valve opened, the slurry solutions is introduced from the packing channel, and flow out of the channel from both ends. As the pores in the frits is much smaller than the resins, the beads are retained within the channel by the frits. With slurry solution pumped in continuously, more and more beads are trapped, and finally, a dense packing bed is formed.

**Section 5. Performance evaluation**

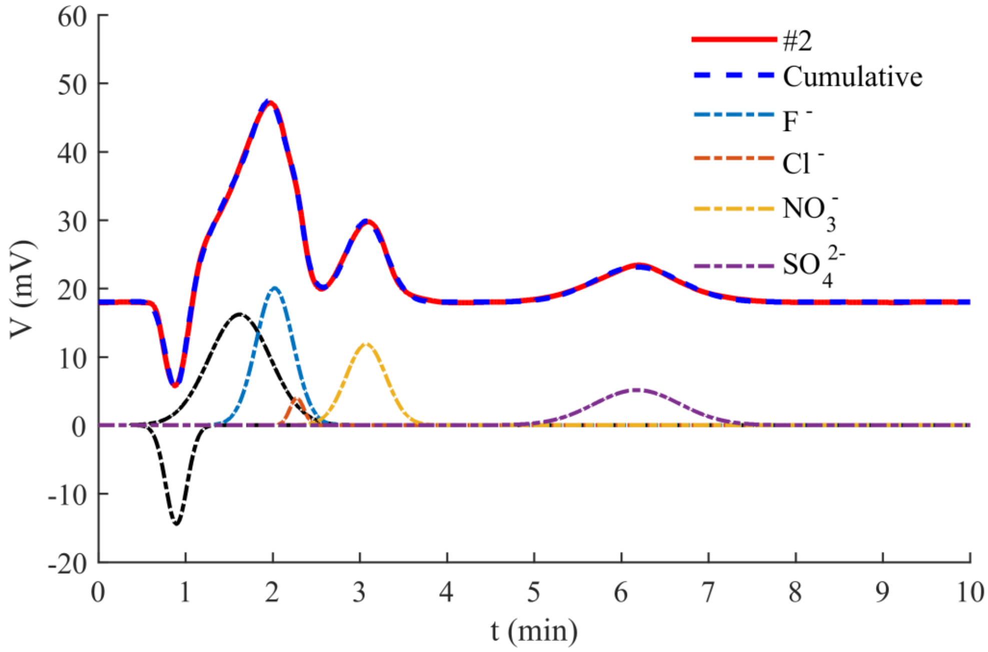


**Figure S6.** Discrimination of the overlapped peaks of $F^{-}$ and $Cl^{-}$ by post-processing.

The post-process is done by using the "*Multiple Peak Fit*" function in OriginPro 9. All peaks are fitted to *Gaussian* type. The negative peak (black) is the water dip. And the first positive peak (black) is related to the resin. The Goodness of Fit ($R^2$) is 0.99936.

**Table S3.** Calculation of chromatograms obtained by chip-IC system at varying flow rate as shown in Figure 5b.

| | *F* (μL/min) | $t_R$ (min) | *H* (mV) | *A* (mV s) | *N* (tp/m) | *R* |
|---|---|---|---|---|---|---|
| $Cl^-$ | 10 | 5.51 | 16.1 | 664.9 | 7182 | |
| | 15 | 3.66 | 15.6 | 476.1 | 5873 | |
| | 20 | 2.74 | 15.3 | 342.3 | 6000 | |
| | 25 | 2.18 | 15.9 | 337.2 | 3891 | |
| $NO_3^-$ | 10 | 8.07 | 10.4 | 486.2 | 11291 | 2.13 |
| | 15 | 5.33 | 10.5 | 365.2 | 8964 | 1.89 |
| | 20 | 4.02 | 10.2 | 301.1 | 7127 | 1.81 |
| | 25 | 3.22 | 9.6 | 240.4 | 7164 | 1.79 |
| $SO_4^{2-}$ | 10 | 15.08 | 10 | 941.3 | 9600 | 3.58 |
| | 15 | 10.2 | 8.7 | 612.1 | 8927 | 3.47 |
| | 20 | 7.66 | 8.7 | 522.6 | 7691 | 3.16 |
| | 25 | 6.19 | 8.2 | 401.9 | 6109 | 2.97 |
